# There is no cheating death: A qualitative study of 4^th^ year medical students’ confrontations with death in their medical curriculum

**DOI:** 10.3205/zma001728

**Published:** 2025-02-17

**Authors:** Alicia Rey, Boris Cantin, Raphaël Bonvin

**Affiliations:** 1 Fribourg Hospital (HFR), Palliative Care Center, Villars-sur-Glâne, Switzerland; 2 University of Fribourg, Faculty of Biology and Medicine, Medical Education Unit, Fribourg, Switzerland

**Keywords:** medical students, attitude to death, medical education, palliative care, qualitative research

## Abstract

**Importance and objective:**

All medical students are confronted with death during their medical curriculum. Despite this, too few qualitative studies have examined this reality’s impact, the support provided by clinical supervisors and the theoretical instruction dedicated to this theme in the university curriculum. This study explores these issues and gives students an opportunity to express themselves, to improve their training and well-being during their pre-graduate.

**Design/method:**

Qualitative study conducted via semi-structured interviews with 4^th^ year medical students at the University of Fribourg engaged in clinical rotations in all the main medical specialties, with in-depth exploration of their experience with death, the support provided by their supervisors and the preparation provided by the university curriculum. A thematic analysis was conducted.

**Findings:**

Five themes emerged: fantasies about death before the encounter, first encounters with death, need for a global approach, impact of clinical supervisors’ skills and denial of death during training. Whether positive or negative, confronting death is often an intense experience for students. They value clinical supervisors who care about how they're feeling, which is all too rare. According to students, the curriculum absolutely needs to be improved to better prepare them for the reality on the ground.

**Conclusion:**

This study highlights the intensity of students’ experiences with death and enables us to propose necessary improvements to their support and training. We believe that students would benefit from a space in which to explore their experiences with death. This would enable them to develop better skills for supporting end-of-life patients and their loved ones, while enhancing their own resources.

## Zusammenfassung

**Bedeutung und Ziel:**

Alle Medizinstudierenden werden während ihres Studiums irgendwann mit dem Thema Tod und Sterben konfrontiert. Dennoch wurden bisher die Auswirkungen dieser Realität, die Unterstützung durch die klinischen Betreuer und die Qualität der theoretischen Ausbildung zu diesem Thema im universitären Lehrplan kaum untersucht. Diese Studie geht der Thematik mit dem Ziel nach, die Vorbereitung auf diese existenzielle Thematik und das Befinden der Studierenden dabei zu verbessern und gibt ihnen die Möglichkeit, sich selbst dazu zu äußern.

**Methode:**

Es handelt sich um eine qualitative Studie mittels semi-strukturierten Interviews mit Medizinstudierenden im 4. Studienjahr an der Universität Fribourg, welche im Rahmen der Rotationspraktika in allen klinischen Hauptfächern tätig sind. Dabei wurden ihre Erfahrungen mit Sterben und Tod, die angebotene Unterstützung durch ihre Supervisoren und die Vorbereitung darauf im Rahmen des Studienplans eingehend untersucht. Es erfolgte eine nach Themenschwerpunkten aufgebaute Analyse.

**Ergebnisse:**

Folgende fünf Themenbereiche wurden behandelt: Fantasien betreffend Sterben und Tod vor dem konkreten Erleben, die erste Begegnung mit dem Tod, die Notwendigkeit eines ganzheitlichen Ansatzes, der Einfluss der Fähigkeiten der klinischen Supervisoren im Umgang mit dem Thema und die Tabuisierung des Todes während der Ausbildung. Ob positiv oder negativ, die Konfrontation mit dem Tod ist in der Regel eine intensive Erfahrung für die Studierenden. Sie schätzen klinische Supervisoren, welche sich um ihr persönliches Befinden kümmern, was leider viel zu selten der Fall ist. Nach Ansicht der Studierenden sollte der Lehrplan unbedingt angepasst werden, um sie besser auf die Realität betreffend die Omnipräsenz von Tod und Sterben in der Medizin vorzubereiten.

**Schlussfolgerung:**

Diese Studie zeigt auf, wie intensiv die Erfahrungen Medizinstudierender mit dem Tod häufig sind und welche Möglichkeiten die Autoren betreffend deren Support und Verbesserungen in der Ausbildung sehen. Wir sind überzeugt, dass die Studierenden davon profitieren würden, wenn ihren Erfahrungen mit dem Tod mehr Raum gegeben wird. Dies würde ihnen ermöglichen, ihre Fähigkeiten für die Begleitung von Sterbenden und deren Angehörigen zu verbessern und gleichzeitig eigene Ressourcen im Umgang mit emotional herausfordernden Situationen zu entwickeln.

## Introduction

Inevitably, all medical students, regardless of future specialty, are confronted with death during their instruction. Whether it’s a patient’s actual death, the risk of death or their own anxieties about the topic, death is omnipresent in the clinical setting. Yet paradoxically, it is rarely addressed in the medical curriculum [1], [2], which has changed little in Switzerland in recent years. To our knowledge few qualitative studies have explored the impacts of students’ early encounters with death [3], [4], [5], [6], [7], [8], [9], [10], [11], [12].

For some authors, this is the result of a medical culture of “death as a failure” rather than the natural outcome of life [11], [12]. While the day-to-day of any caregiver includes death and the fear that it instills, students often find their first experiences of death extremely intense [8], [10], [11], [13], [14], [15]. All too often, students’ early experiences are traumatic, particularly when clinical supervisors fail to provide the necessary support [11], [16]. This solitary “baptism by fire”, endured with little theoretical and practical instruction, poses a significant risk of suffering in students [9], [11]. 

Yet students value instruction on end-of-life issues [17], [18], want more of it [19], [20] and rarely feel that they have had enough [11], [21], [22], [23], [24], [25], [26], [27].

These findings and the important lack of detailed data from students’ point of view on this crucial topic prompted this study, whose goal is to provide a better understanding of students’ experiences and needs with respect to death and the issues that surround it, both in terms of clinical practice and the development of their medical identity.

## Methodology

### Study design and recruitment

This qualitative study uses a thematic analysis approach with a constructivist orientation and the ontological assumption that students can have a wide variety of experiences on this topic that are often intense and under-explored and require in-depth exploration. Given the study’s design, the local ethics commission decided that the need for approval was waived. This study followed the Standards for Reporting Qualitative Research (SRQR) reporting guideline [28] and the Consolidated criteria for REporting Qualitative studies (COREQ) [29].

Purposive sampling was performed to reflect the diversity of the experiences. AR and BC presented the research topic in person at the beginning of 2021 to fourth-year students of a six-year undergraduate medical program at the University of Fribourg who were invited to participate.

All participants signed a written consent form.

### Data collection

Semi-structured face-to-face in-depth individual interviews were conducted by the principal investigator (AR, palliative care specialist and active in medical education at the University with no involvement in student assessment) at the palliative care center during fourth-year clinical rotations in the main medical specialties. Based on the existing literature and a discussion among the three investigators, the interview guide (see table 1 (Tab. 1)) focused on three areas: students’ experiences of confronting death in a clinical setting, the support their clinical supervisors provided, and preparation for these experiences via the curriculum before their clinical rotations. The interview guide was subsequently adapted as the interviews progressed.

The interviews were audio-recorded, anonymized (using aliases) and transcribed externally (verified by AR).

### Data analysis

The three investigators (AR, RB expert in medical education and BC expert in palliative care, the last two with prior experience in qualitative research with publications) independently analyzed the interviews via inductive thematic analysis [30], [31]. First, they performed iterative readings of the transcripts to become familiar with the data and structure it as codes by segmenting information. These initial codes were then analytically organized in subcategories and categories to develop themes of interest consistent with the dataset (verification performed by returning to source data by the three investigators). Lastly, the three authors discussed their codes, categories and themes on several occasions to reach a consensus and illustrated them with relevant quotes. This iterative analysis by investigators from different backgrounds broadens perspectives and reinforces the analysis’s credibility and validity. They continually reflected on their own point of view and how it might influence the analysis and interpretation.

## Results

The participants – 10 women and 4 men, all students from the same cohort – were interviewed from March to May 2021; interviews lasted an average of 60 minutes. The recruitment ended with 14 participants once data analysis of new interviews yielded no additional codes (saturation).

The thematic analysis yielded five themes (see table 2 (Tab. 2)).

### Fantasies about death before entering the clinical setting: A “sword of Damocles”

Students often anticipate their first experiences confronting death in the clinical setting, sometimes with curiosity or respect, sometimes with apprehension. Many wondered how they would react to a patient’s death, some fearing being overcome with emotion.

*“Until I experienced it, I felt a sword of Damocles over my head, something to be dreaded.” (Valentin)*

They often share their apprehensions with their peers, including that they will not know how to behave prior to the death and that they may even be responsible for it.

### First encounters with death: powerful, thought-provoking experiences

Far from being gloomy or terrifying, some experiences are described as beautiful, powerful and rewarding.

They believe that death is part of being a doctor and part of life. Provided that they are well experienced, encounters with death in the clinical setting are valued as important steps in the development of their medical identity, easier to experience as a trainee than as a young doctor with more responsibilities. This experience can be a source of questioning about the place death holds in medicine and about patients’ needs.

*“I find that, during our studies, we focus on the good, but the good from whose perspective? What I learned is that the other person’s needs aren’t necessarily what we’d expect [...] it really made me think.” (Valentin)*

Supporting the patient until death, the authenticity of the therapeutic bond and the care provided when curative possibilities have been exhausted are described as essential.

The death pronouncement is perceived not as a simple administrative task, but as a special moment between patient and physician.

Regarding communicating with patients and their loved ones, the participants shared their fears of hurting people with clumsy words or of feeling helpless.

They also spoke of experiences of sharing that were formative and meaningful. Discussions can be so intense that they wonder about the distance between doctors and patients. Participants talk about their need to keep feeling, while shielding themselves from overwhelming emotions, to maintain both personal balance and professionalism. 

Other experiences are more difficult and often have more to do with what surrounds death than with the moment of death itself. Interactions with patients’ loved ones are touching moments that evoke empathy and sometimes a feeling of identification. 

*“Often, the hardest part is the family, seeing the looks on their faces and sensing their emotions. I know that it is going to affect me.” (Paul)*

Speaking with their peers, clinical supervisors and relatives is an important resource for adapting. The students also talked about personal reflection, the meaning attributed to the experiences, humor, leisure, repression and rationalization. The death experience seems to stay with students well beyond the hospital walls.

*“My main resource was my roommates [...] I think I’ve told them about most of the times when a patient died. At least I had told someone... who understands.” (Olivia)*

Some mention having to confront their own mortality, a potential source of anxiety, but also that they have significant positive and enriching existential thoughts. They mention that this aspect, if poorly experienced and not necessarily acknowledged, could explain some relationship problems observed among clinical supervisors, as well as the instances of therapeutic overkill also noted.

*“Fear of getting it wrong, of not knowing how to do it. [...] Maybe also personally being afraid. Not having given death much thought, how would I feel? How can you understand a patient if you’ve never asked yourself that question?” (Celine)*

Awareness of our own mortality can be meaningful, bringing us to appreciate life more, reassess our priorities and grow personally and professionally.

### Need for a global, person-centered approach to avoid fragmenting the patient

The concepts of quality of life and holism are described as being fundamental, but they are poorly explored and integrated in the clinical setting. 

Questions arise about the point or futility of certain treatments; given the degree of perceived suffering, some students even speak of “therapeutic overkill.” They complain that some clinical supervisors appear to see death as a failure to be avoided at all costs, displaying a sense of medical omnipotence, to the detriment of patients.

Students observed a failure to identify end-of-life situations, with negative consequences on the support patients and their families receive.

*“He was no longer responding or moving. It was obvious to me that he was on his deathbed. And the doctor was still thinking, “I want to save this patient and if we’re lucky, he’ll thank us for it.” It’s sad. I actually thought to myself, “Ah, so this is what therapeutic overkill looks like.”” (Mary)*

Without minimizing the satisfaction of solving medical problems, the students value their talks with patients for whom a cure or even more life isn’t possible or wanted.

They mention the importance of respecting patients’ autonomy, knowing about their deepest values and fully understanding their situation to best support them as they face a grave illness and death. 

They lament the lack of exploration of patients’ experiences and cite patient fragmentation and medical hyper-specialization as potential barriers to quality care. This can lead to a painful disconnect between the medical perspective and the needs of patients.

They described a sense of near dehumanization, as if it were sometimes more a matter of treating pathologies and organs than people with a life story, values and plans. In the face of death, we sometimes rediscover this sense of wholeness, as though it re-humanizes the patient.

*“It seemed to me that they were being treated not as people, but as machines we want to get working again. But then suddenly, that patient who died became a real person again. That was touching. First, he was an object rather than a subject, but after death he became human again.” (Christine)*

### The impact of clinical supervisors’ skills in dealing with death and supporting students

Students report big differences in clinical supervisors’ attitudes toward death. 

Some clinical supervisors are described as being involved, empathetic and equipped to support patients, which students who cite them as examples appreciate. But others seem uncomfortable with death and the patient’s emotions, sometimes much more so than the students themselves.

*“You can tell they’re not comfortable with the subject. They don’t know what to say, so they avoid it. I might not say the right thing, but at least I would say something. They just say NOTHING! That’s so frustrating!” (Céline)*

Some describe the doctor’s lack of interest in the patient when death seems inevitable, as if it were someone else’s problem.

The support clinical supervisors provide students with also varies. They speak about positive experiences where they felt supported and about the need to discuss their experiences with death in a climate of trust.

*“What it did for me was... huge, really. He prepared me, took the time to speak with me and reassure me. He dug a bit deeper: “How did you feel?” It did me so much good. While he didn’t really provide answers, he validated what I had felt, without judging.” (Valentin)*

Because they are not always aware of their own need to share, they appreciate when clinical supervisors take the initiative.

*“Talking about it was what made me realize that I had needed to talk about it. It was only later that I realized that I had come face to face with death.” (Maxime)*

Yet their experiences too often remain unexplored, despite some of their early experiences being brutal.

*“I exited the hospital and just bawled my eyes out. There were lots of people around me... doctors, families passing by... Nobody reacted. It was really shocking.” (Blue)*

### Denial of death during training

The students share their need to address the subject of death and the emotions that come with it, as they feel unprepared to deal with it in the clinical setting.

While valuing concrete experiences, they suggest how training could be improved. With the curriculum described as focusing primarily on differential diagnosis, solution-finding and technicality, they stress the importance of breaking the taboo surrounding death.

They feel the need to be clearly warned that they will have to face death and that it may summon up powerful emotions.

## Discussion

These first experiences of confrontation with death occur early and are often very intense and a source of reflection for students. Support from supervisors is therefore extremely important and could be improved, as could teaching on this subject during studies. These points will be discussed here.

Although sometimes unsettling or painful, most students appreciate these discussions and the connection that can develop between patients confronted with death and their physicians. Yet they note that these topics are not really covered in the theoretical curriculum or in clinical practice, a finding that is in line with the existing literature [8], [25]. They bemoan a certain dehumanization of medicine – while not systematic, it is a sad reality.

It seems essential for someone going into healthcare to reflect on life and death. We believe it is necessary to foster and strengthen students’ “people skills” to improve their ability to support patients confronted with death. 

The personal, existential component of encounters with death is mentioned, with thoughts on the meaning of life, as well as the taboo surrounding death. Encountering death in a clinical setting does not always go hand in hand with being able to discuss it comfortably.

It is worth noting that students confronted with death for the first time in their careers seem to experience very powerful emotions, while also reflecting rather deeply on the matter. At the same time, they are aware of a degree of emotional blunting among some of their senior colleagues. What assumptions could be made about their experience with death as they evolve over time? Over the long term, being repeatedly confronted with suffering and death might be too painful to experience fully. The students raise the notion of distance, whether positive (protective) or negative (harmful defense mechanism). In our opinion, this emotional distancing is not without danger: as the emotions have not been acknowledged, the distancing is often unconscious. This might be due to a medical culture that rarely legitimizes the professional’s emotional experience and sometimes even goes as far as to consider it a weakness unworthy of a physician. Most students we met with disagree with this view and, to the contrary, appreciate having their experiences explored and validated by their clinical supervisors.

Regarding the role of supervisors, we note that the most positive accounts share not only the very human support provided to the patient, but also the clinical supervisor’s consideration of the student’s emotional experience. Support for the student is indeed an essential resource when dealing with death in a clinical setting [4], [5], [6], [9], [32], [33], [34]. Even when the situation does not call for an in-depth discussion, students appreciate having a climate of openness and clinical supervisors who initiate the discussion, especially given that students can be hesitant about raising this delicate subject on their own. The critical importance of this approach can be understood by comparing the various narratives and how they affect the role model. How the clinical supervisor supports a student’s encounter with death can make the difference between a long-lasting trauma, sometimes going so far as to call into question one’s choice of profession, and an enriching experience with a consequent impact on the development of the student’s medical identity.

Regarding training, we believe that better preparing students for their encounters with death would have a significant positive impact on the care afforded patients and their loved ones, not to mention students’ well-being. Some authors have already demonstrated that young physicians do not feel sufficiently prepared at the start of their careers [35].

As far as theoretical training is concerned, both quantitative and qualitative progress seems possible, an observation also supported by the World Health Organization [36]. This topic is rarely discussed, and when it is, it is almost exclusively during the rare course given on palliative care, as if this setting was the only one in which death occurs. In truth, death occurs everywhere in medicine, much more often than students expect. We believe that this cross-cutting issue would benefit from clearer messages, as shown in table 2 (Tab. 2). In our view, this should be a cross-cutting theme that is addressed in all medical specialties where death may be an outcome. Not mentioning it as one possible outcome among others gives students a partial and distorted image of clinical reality.

In the different Swiss medical schools, questions about life and death are mainly addressed in palliative care and ethics courses, which account for a few hours of the entire curriculum (depending on the university), with the first contact with patients facing death taking place in 4^th^ year at the latest.

Although vital, theoretical learning cannot replace personal experience. We could consider providing opportunities to share experiences, emotions and thoughts with identified senior physicians prepared to play an active role.

Simulations that allow students to practice their communication skills regarding end-of-life issues including debriefing that stimulates reflection on the subject of death also seem effective [27], [37], [38]. Self-awareness and self-reflection are also considered important elements that can be developed to support self-care when encountering death [33].

Opportunities for students to speak with patients (or their loved ones) confronted with death could also be an interesting option, as would palliative care immersion courses [39], [40], [41], [42].

The students’ rich narrative clearly shows that the support they receive needs to be improved (see table 3 (Tab. 3)).

This is a delicate issue in which clinical supervisor’s experience does not always equate with being comfortable with death. Admitting one’s own vulnerability can be difficult, especially in an environment where a physician’s emotions are sometimes seen as weaknesses. However, we believe that being able to speak openly about death would benefit everyone. Discussing it among professionals could be a first step toward greater ease talking about it with patients, who overwhelmingly appreciate feeling that they are welcome to speak freely on this issue [43].

### Strengths and limitations

To our knowledge, this is the first qualitative study to explore all at once, through in-depth interviews, the experiences of medical students confronted with death, the support they receive and the preparation they receive through theoretical training on this issue.

Voluntary participation can lead to selection bias (ease of expression about death and emotions, interest). Also, the participants hail from the same academic program. To gain a deeper understanding of the topic, further research is needed.

## Conclusion

For better or for worse, the inevitable experiences of students confronted with death in a professional context appear to be very intense. While potentially formative and enriching, they can also be painful and even traumatic. Receiving careful, individualized support from clinical supervisors is one of the key elements that make these experiences positive for students, a finding consistent with those of other studies [8], [10]. Unfortunately, this does not always occur and the accounts of many students have convinced us that improvements are needed. Students also appreciate having the issues of death, support for patients and their loved ones, and their own emotions addressed as part of the university curriculum – this observation provides some insight into possible improvements in training. This study highlights the importance of providing an open and caring space in which students can express their feelings. We are convinced that it is also the responsibility of supervisors to offer them this space, in both an informal and structured way. This contributes to their quality of life during their studies and can have a significant impact on the development of their professional identity.

We believe it is essential to reconsider how we, as health professionals, view death. Often perceived as a failure, death can create a feeling of helplessness among the physicians who have trouble accepting it, as they consider their mission to be saving lives at all costs. We sometimes seem to have forgotten that, despite incredible advances in medicine, death is still a part of life that concerns us all as humans. So as physicians, what kind of space are we prepared to create for death?

## Notes

### Author contributions

Alicia Rey had full access to all the data in the study and takes responsibility for the integrity of the data and the accuracy of the data analysis.

- Concept and design: Rey, Cantin, Bonvin
- Acquisition, analysis, or interpretation of data: Rey, Cantin, Bonvin
- Drafting of the manuscript: Rey
- Critical revision of the manuscript for important intellectual content: Cantin, Bonvin
- Administrative, technical, or material support: Bonvin
- Supervision: Cantin, Bonvin

### Authors’ ORCIDs

- Alicia Rey: [0009-0006-9116-9056]
- Raphaël Bonvin: [0000-0001-9322-2494]

### Data management and sharing

To protect participants’ privacy, transcripts will not be shared. However, supplementary material may be made available from the first author upon request.

## Acknowledgements

The authors would like to thank all the participants, David Shotlander for the professional translation and Prof. Christine Cedraschi for her help.

## Competing interests

The authors declare that they have no competing interests. 

## Figures and Tables

**Table 1:**
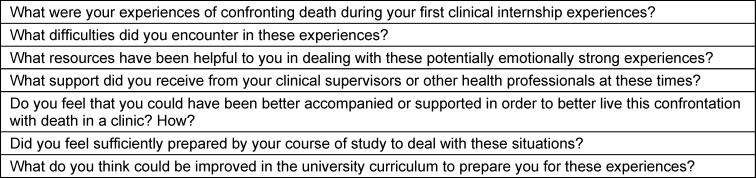
Interview guide

**Table 2:**
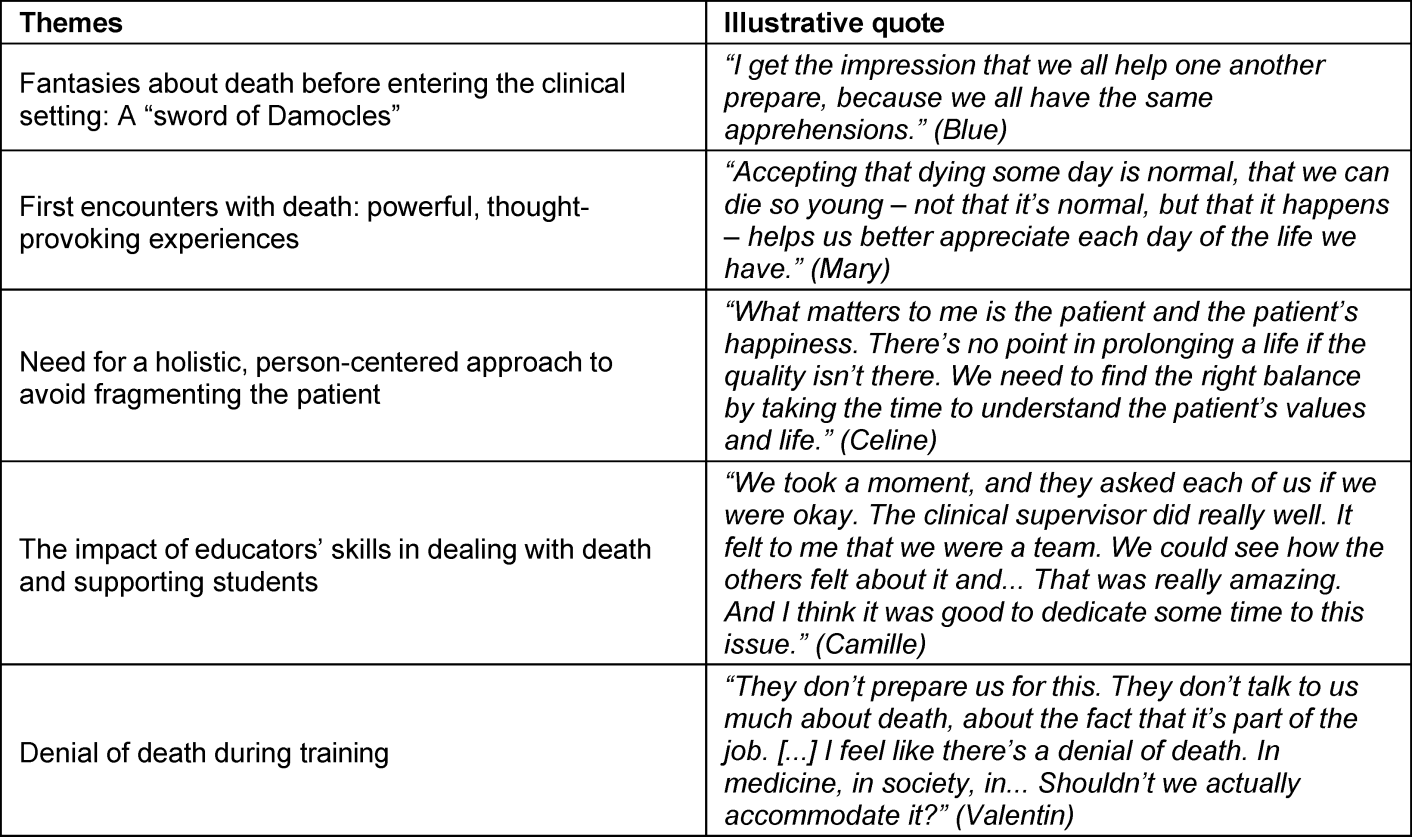
Illustrative quotes from the different themes

**Table 3:**
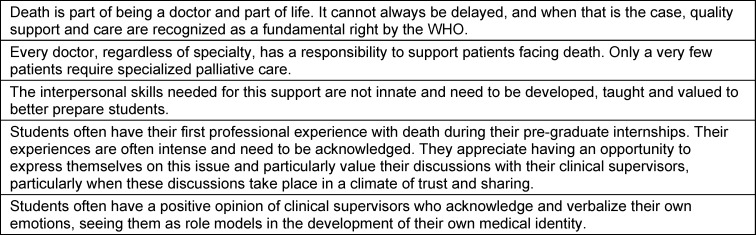
Key messages about death in the clinical setting, inspired by student accounts and the literature [10], [36], [44], [45], [46], [47]

## References

[1] Pereira J, Pautex S, Cantin B, Gudat H, Zaugg K, Eychmuller S, Zulian G (2008). Palliative care education in Swiss undergraduate medical curricula: a case of too little, too early. Palliat Med.

[2] Wass H (2004). A perspective on the current state of death education. Death Stud.

[3] Batley NJ, Bakhti R, Chami A, Jabbour E, Bachir R, El Khuri C, Mufarrij AJ (2017). The effect of patient death on medical students in the emergency department. BMC Med Educ.

[4] Boland JW, Dikomitis L, Gadoud A (2016). Medical students writing on death, dying and palliative care: a qualitative analysis of reflective essays. BMJ Support Palliat Care.

[5] Kelly E, Nisker J (2010). Medical students’ first clinical experiences of death. Med Educ.

[6] Pessagno R, Foote CE, Aponte R (2014). Dealing with death: medical students’ experiences with patient loss. Omega (Westport).

[7] Ratanawongsa N, Teherani A, Hauer KE (2005). Third-year medical students’ experiences with dying patients during the internal medicine clerkship: a qualitative study of the informal curriculum. Acad Med.

[8] Rhodes-Kropf J, Carmody SS, Seltzer D, Redinbaugh E, Gadmer N, Block SD, Arnold RM (2005). “This is just too awful; I just can’t believe I experienced that...”: medical students’ reactions to their “most memorable” patient death. Acad Med.

[9] Smith-Han K, Martyn H, Barrett A, Nicholson H (2016). That’s not what you expect to do as a doctor, you know, you don’t expect your patients to die." Death as a learning experience for undergraduate medical students. BMC Med Educ.

[10] Trivate T, Dennis AA, Sholl S, Wilkinson T (2019). Learning and coping through reflection: exploring patient death experiences of medical students. BMC Med Educ.

[11] Wear D (2002). “Face-to-face with It”: medical students’ narratives about their end-of-life education. Acad Med.

[12] Felber SJ, Guffi T, Brem BG, Schmitz FM, Schnabel KP, Guttormsen Schär S, Eychmüller S, Zambrano SC (2024). Talking about dying and death: Essentials of communicating about approaching death from the perspective of major stakeholders. Palliat Support Care.

[13] Barton D (1972). The need for including instruction on death and dying in the medical curriculum. J Med Educ.

[14] Santen S, Bartlett E, Mukhtar N (2007). First death. Acad Emerg Med.

[15] Williams CM, Wilson CC, Olsen CH (2005). Dying, death, and medical education: student voices. J Palliat Med.

[16] Haglund ME, aan het Rot M, Cooper NS, Nestadt PS, Muller D, Southwick SM, Charney DS (2009). Resilience in the third year of medical school: a prospective study of the associations between stressful events occurring during clinical rotations and student well-being. Acad Med.

[17] Hegedus K, Zana A, Szabó G (2008). Effect of end of life education on medical students’ and health care workers’ death attitude. Palliat Med.

[18] Kaye J, Gracely E, Loscalzo G (1994). Changes in students’ attitudes following a course on death and dying: a controlled comparison. J Cancer Educ.

[19] Hesselink BA, Pasman HR, van der Wal G, Soethout MB, Onwuteaka-Philipsen BD (2010). Education on end-of-life care in the medical curriculum: students’ opinions and knowledge. J Palliat Med.

[20] Jiang X, Liao Z, Hao J, Guo Y, Zhou Y, Ning L, Baio J, Zhang P, Tang C, Zhao X, Guo H (2011). Palliative care education in China: insight into one medical university. J Pain Symptom Manage.

[21] Black D, Hardoff D, Nelki J (1989). Educating medical students about death and dying. Arch Dis Child.

[22] Buss MK, Marx ES, Sulmasy DP (1998). The preparedness of students to discuss end-of-life issues with patients. Acad Med.

[23] Tan A (2013). Medical students and dying patients. Virtual Mentor.

[24] MacKenzie AR, Lasota M (2020). Bringing Life to Death: The Need for Honest, Compassionate, and Effective End-of-Life Conversations. Am Soc Clin Oncol Educ Book.

[25] Cavalcinta de Siqueira ME, Mergulhão LM, Sant'Anna Pires RF, de Pádua Walfrido Jordán A, Barbosa LN (2022). Attitude to death and opinion of medical students on the top training. Rev Bras Educ Med.

[26] Keleş Ş, Gül Ş, Yıldız A, Karabulut SD, Eren H, İskender MD, Baykara ZG, Yalım NY (2023). Ethical Discourse of Medical Students on the Phenomenon of Death: A Qualitative Study. Omega (Westport).

[27] Wells G, Llewellyn C, Hiersche A, Minton O, Barclay D, Wright J (2024). Care of the dying - medical student confidence and preparedness: mixed-methods simulation study. BMJ Support Palliat Care.

[28] O’Brien BC, Harris IB, Beckman TJ, Reed DA, Cook DA (2014). Standards for reporting qualitative research: a synthesis of recommendations. Acad Med.

[29] Tong A, Sainsbury P, Craig J (2007). Consolidated criteria for reporting qualitative research (COREQ): a 32-item checklist for interviews and focus groups. Int J Qual Health Care.

[30] Braun V, Clarke V (2006). Using thematic analysis in psychology. Qual Res Psychol.

[31] Creswell JW (2007). Qualitative inquiry & research design: choosing among five approaches.

[32] Ho CY, Kow CS, Chia CH, Low JY, Lai YH, Lauw SK, How AE, Tan LH, Ngiam XL, Chan NP, Kuek TY, Kamal NH, Chia JL, Bin Hanifah Marican Abdurrahm A, Chiam M, Ong YT, Chin AM, Toh YP, Manson S, Krishna LK (2020). The impact of death and dying on the personhood of medical students: a systematic scoping review. BMC Med Educ.

[33] Felber SJ, Zambrano SC, Guffi T, Schmitz FM, Brem BG, Schnabel KP, Guttormsen S, Eychmüller S (2024). How to talk about dying? The development of an evidence-based model for communication with patients in their last days of life and their family caregivers. PEC Innov.

[34] Parry M, Jones B, Churcher C (2022). End-of-life simulation: a cross-field evaluation in an undergraduate nursing programme. Int J Palliat Nurs.

[35] Gibbins J, McCoubrie R, Forbes K (2011). Why are newly qualified doctors unprepared to care for patients at the end of life?. Med Educ.

[36] World Health Organization (2020). Palliative care.

[37] Bansal A, Monk A, Norman M, Fingas S (2020). Improving medical students’ confidence in end-of-life consultations. Clin Teach.

[38] Nordström A, Fjellman-Wiklund A, Grysell T (2011). Drama as a pedagogical tool for practicing death notification-experiences from Swedish medical students. BMC Med Educ.

[39] Shunkwiler SM, Broderick A, Stansfield RB, Rosenbaum M (2005). Pilot of a hospice-based elective to learn comfort with dying patients in undergraduate medical education. J Palliat Med.

[40] Mott ML, Gorawara-Bhat R, Marschke M, Levine S (2014). Medical students as hospice volunteers: reflections on an early experiential training program in end-of-life care education. J Palliat Med.

[41] Hawes AM (2020). The Spectrum of Deaths Encountered by a Young Learner. JAMA.

[42] Sampath R (2020). Always More to Be Done. JAMA.

[43] Zweers D, de Graeff A, Duijn J, de Graaf E, Witteveen PO, Teunissen SC (2019). Patients’ Needs Regarding Anxiety Management in Palliative Cancer Care: A Qualitative Study in a Hospice Setting. Am J Hosp Palliat Care.

[44] Gott M, Frey R, Raphael D, O’Callaghan A, Robinson J, Boyd M (2013). Palliative care need and management in the acute hospital setting: a census of one New Zealand Hospital. BMC Palliat Care.

[45] Meffert C, Rücker G, Hatami I, Becker G (2016). Identification of hospital patients in need of palliative care--a predictive score. BMC Palliat Care.

[46] To TH, Greene AG, Agar MR, Currow DC (2011). A point prevalence survey of hospital inpatients to define the proportion with palliation as the primary goal of care and the need for specialist palliative care. Intern Med J.

[47] Morin L, Aubry R, Frova L, MacLeod R, Wilson DM, Loucka M, Csikos A, Ruiz-Ramons M, Cardenas-Turanzas M, Rhee YJ, Teno J, Öhlén J, Deliens L, Houttekier D, Cohen J (2017). Estimating the need for palliative care at the population level: A cross-national study in 12 countries. Palliat Med.

